# Protection Strategy against an Epidemic Disease on Edge-Weighted Graphs Applied to a COVID-19 Case

**DOI:** 10.3390/biology10070667

**Published:** 2021-07-15

**Authors:** Ronald Manríquez, Camilo Guerrero-Nancuante, Carla Taramasco

**Affiliations:** 1 Laboratorio de Investigación Lab[e]saM, Departamento de Matemática y Estadística, Universidad de Playa Ancha, Valparaíso 2340000, Chile; 2 Escuela de Enfermería, Universidad de Valparaíso, Viña del Mar 2520000, Chile; 3 Escuela de Ingeniería Civil Informática, Universidad de Valparaíso, Valparaíso 2340000, Chile; 4 Centro Nacional de Sistemas de Información en Salud, Santiago 8320000, Chile

**Keywords:** edge-weighted graph, SIR model, graph protection, COVID-19

## Abstract

**Simple Summary:**

Infectious diseases have been part of human history. Countless epidemics have produced high mortality rates in vulnerable populations. With the understanding of the spread of these types of diseases, population groups have been able to adapt and better cope with infections. Given the COVID-19 pandemic, one of the strategies used is the modeling of infectious diseases with the aim of establishing protection measures for people and stopping the spread of the epidemic. Our study evaluates protection strategies through infectious disease modeling with COVID-19 data in a commune in Chile. The results of the simulations indicate that the model generates important protection for the population by recognizing the super-propagating people (bridge nodes). This type of protection can be key in the fight against COVID-19.

**Abstract:**

Among the diverse and important applications that networks currently have is the modeling of infectious diseases. Immunization, or the process of protecting nodes in the network, plays a key role in stopping diseases from spreading. Hence the importance of having tools or strategies that allow the solving of this challenge. In this paper, we evaluate the effectiveness of the DIL-W${}^{\alpha }$ ranking in immunizing nodes in an edge-weighted network with 3866 nodes and 6,841,470 edges. The network is obtained from a real database and the spread of COVID-19 was modeled with the classic SIR model. We apply the protection to the network, according to the importance ranking list produced by DIL-W${}^{\alpha }$, considering different protection budgets. Furthermore, we consider three different values for α; in this way, we compare how the protection performs according to the value of α.

## 1. Introduction

Infectious diseases have been the focus of multiple fields of research. In public health and epidemiology, efforts are directed at establishing transmission dynamics, the characteristics of infectious agents, and the populations most affected by pathogens, among others, which are of high importance for science [1]. In recent decades, research on infectious diseases has involved the application of complex theories from mathematics and engineering. In particular, the use of network models has allowed explanations of the spread of diseases from infected people (nodes) and their links with others (edges) [2].

Network models establish the connection between population groups, which is useful not only in the field of public health or epidemiology, but also in engineering and social sciences [3] (see [4,5,6,7,8]). In the health field, being a theoretical approach, the importance of recognizing the complexities of community structures has been discussed in order to understand social dynamics in the spread of infectious diseases [9]. For example, Magelinski et al. developed a model to estimate the role played by certain nodes in community structures [10], while Ghalmane et al. included the dimension of centrality in complex networks [11].

Regarding the COVID-19 pandemic, a series of models have been developed which allow the projection and establishment of the progress of this infectious disease based on the data available from different information sources (see, for instance, [12]). In this sense, Manríquez et al. propose the use of weighted graphs at the edges, giving the network model, from a stochastic approach, the possibility of identifying the most important variables in the spread of COVID-19 with real data in a city in Chile [13]. The same authors, in order to classify the importance of the nodes, propose the generalization of the measure of importance of the line with the use of degree of centrality (DIL-W${}^{\alpha }$), improving the understanding of the network from a local perspective [14].

On the other hand, the same network models have served to search for protection strategies for populations, generally related to the processes of immunization or isolation of people (see [15,16,17,18,19,20]).

Scientific evidence supports immunization strategies being useful in both homogeneous and heterogeneous networks [21]. The strategies seek, first, to develop a dynamic experimental model that includes the classic compartmental systems in epidemiology (SIR, SEIR, SIS, among others) and then to establish immunization measures across the nodes. Immunization measures can be applied in static and dynamic networks, in a random or targeted immunization [22].

Random immunization refers to random strategies, without determining a particular population in the protection process [23]. In contrast, targeted immunization recognizes nodes with a higher degree of connection with other nodes [24]. Nian et al. conclude, through computational simulations in a free scale graph (Model BA), that targeted immunization is more effective than randomized [21]. In the same direction, Wang et al. conclude that, even if the immunization strategy is imperfect or incomplete, it manages to generate positive impacts on the protection of the network [25]. Another investigation by Xia et al. determined that targeted immunization in two rounds of selection provides a greater protective effect compared to progressive strategies [24].

According to the above, a study conducted by Ghalmane et al. proposed an immunization strategy considering the influence of the nodes, the number of communities and the links between them [26]. Along the same lines, Gupta et al. analyzed the importance of protection strategies using information from community networks [27]. Since edge-weighted graphs have been an important way of understanding epidemic diseases, Manríquez et al. measure the effectiveness of DIL-W${}^{\alpha }$ with the recognition of bridge nodes. The effectiveness of DIL-W${}^{\alpha }$ is high compared to other proposals on four real networks [28].

For all the above, studies that include immunization measures together with community/local network models are highly relevant for public health, both for establishing measures to mitigate epidemic diseases and for the development of vaccination optimization policies to more quickly achieve herd immunization and, therefore, overcome diseases such as COVID-19. This is supported by Zhao et al., who argue that this framework allows for the optimization of immunization resources [29].

This study aims to analyze the protection effect against COVID-19 using the DIL-W${}^{\alpha }$ ranking with real data from a city in Chile (Olmué-City), obtained from the Epidemiological Surveillance System of the Ministry of Health of Chile, from which we obtain an edge-weighted graph, denoted by $G_{E}$, according to the method proposed in [13]. We apply the protection to the $G_{E}$ network, according to the importance ranking list produced by DIL-W${}^{\alpha }$, considering different protection budgets. For the ranking DIL-W${}^{\alpha }$, we consider three different values for α; they are 0, 0.5 and 1. In this way, we compare how the protection performs according to the value of α. We use a graph-based SIR model, namely, each individual is represented by a vertex in $G_{E}$. At time *t*, each vertex $v_{i}$ is in a state $v_{i}^{t}$ belonging to S={0,1,−1}, where 0,1 and −1 represent the three discrete states: Susceptible (S), Infected (I) and Recovered or Removed (R). Five hundred simulations were performed on $G_{E}$; the initial population contains one infected node and all the simulations, considering $\delta =\frac{1}{15}$ (recovered rate).

This paper is organized as follows: Section 2 contains generalities about graph theory, includes a graph from a database, and the DIL-W${}^{\alpha }$ ranking is explained. In Section 3, we obtain the graph from a real database from a city in Chile (Olmué-City), and we set the protection strategy. In Section 4, the results of the study are presented. Section 5 provides a discussion of the results and potentialities of the method used. Finally, Section 6 provides the conclusions.

## 2. Basic Definitions

In this section, we establish the definitions and elements used throughout this paper. We summarize the symbols and notations in Table 1.

### 2.1. Graphs

The following definitions come from [30,31].

**Definition** **1.** *A graph G is a finite nonempty set V of objects called vertices, together with a possibly empty set E of 2-element subsets of V called edges.*

To indicate that a graph *G* has vertex set *V* and edge set *E*, we write G=(V,E). If the set of vertices is $V=\{v_{1},v_{2},\ldots ,v_{n}\}$, then the edge between vertex $v_{i}$ and vertex $v_{j}$ is denoted by $e_{ij}$.

If $e_{ij}$ is an edge of *G*, then $v_{i}$ and $v_{j}$ are adjacent vertices. Two adjacent vertices are referred to as neighbors of each other. The set of neighbors of a vertex *v* is called the open neighborhood of *v* (or simply the neighborhood of *v*) and is denoted by N(v). If $e_{ij}$ and $e_{jk}$ are distinct edges in *G*, then $e_{ij}$ and $e_{jk}$ are adjacent edges.

**Definition** **2.** *The number of vertices in a graph G is the order of G and the number of edges is the size of G.*

**Definition** **3.** *The degree of a vertex v in a graph G, denoted by deg(v), is the number of vertices in G that are adjacent to v. Thus, the degree of v is the number of vertices in its neighborhood N(v).*

**Definition** **4.** *Let G be a graph of order n, where $V\left(G\right)=\left\{v_{1},v_{2},\ldots ,v_{n}\right\}$. The adjacency matrix of G is the n×n zero-one matrix $A\left(G\right)=\left[a_{ij}\right]$, or simply $A=[a_{ij}]$, where*

$$a_{ij}=\left\{\begin{array}{cc} 1 & if\;\;e_{ij}\in E\left(G\right)\\ 0 & if\;\;e_{ij}\notin E\left(G\right). \end{array}\right.$$

On the other hand, an important generalization of the simple graph consists of the definition of a weighted graph, more specifically an edge-weighted graph. Informally, an edge-weighted graph is a graph whose edges have been assigned a weight.

**Definition** **5.** *An edge-weighted graph is a pair (G,W), where G=(V,E) is a graph and W:E→R is a weight function. If $e_{ij}\in E$ then $W\left(e_{ij}\right)=w_{ij}$.*

**Definition** **6.** *The strength of a vertex $v_{i}$, denoted by $S(v_{i})$, is defined as the sum of the weights of all edges incident to it, this is to say,*

$$S\left(v_{i}\right)=\sum_{v_{j}\in N\left(v_{i}\right)}w_{ij}.$$

The following definition comes from [32].

**Definition** **7** (Degree centrality [32])**.** *The degree centrality of $v_{i}\in V$ of an edge-weighted graph (G,w), denoted by $C_{D}^{w\alpha }\left(v_{i}\right)$, is defined as*

(1) $$\begin{array}{cc} C_{D}^{w\alpha }\left(v_{i}\right) & =deg{\left(v_{i}\right)}^{\left(1-\alpha \right)}\cdot S{\left(v_{i}\right)}^{\alpha }, \end{array}$$

*where α∈[0,1].*

The parameter α is called the *tuning parameter*. Notice that, when α=0, then $C_{D}^{w\alpha }\left(v_{i}\right)=deg\left(v_{i}\right)$ and, when α=1, then $C_{D}^{w\alpha }\left(v_{i}\right)=S\left(v_{i}\right)$.

### 2.2. DIL-W${}^{\alpha }$ Ranking

We briefly describe the DIL-W${}^{\alpha }$ ranking in this Section. The DIL ranking is a tool for evaluating the node importance based on degree and the importance of lines (DIL) proposed by Liu et al. in [33] for an undirected and unweighted network. Recently, Manríquez et al. in [14] propose DIL-W${}^{\alpha }$ rank. This ranking method of node importance for undirected and edge-weighted is a generalization of the measure of line importance (DIL) based on the centrality degree (Definition 7) proposed by Opsahl in [32].

The following comes from [14].

Let us consider an undirected weighted graph (G,w) with G=(V,E) and $V=\{v_{1},v_{2},\ldots ,v_{n}\}$.

**Definition** **8** (Importance edge [14])**.** *The importance of an edge $e_{ij}\in E$, denoted by $I^{\alpha }\left(e_{ij}\right)$, is defined as*

$$\begin{array}{cc} I^{\alpha }\left(e_{ij}\right) & =\frac{\left(C_{D}^{w\alpha }\left(v_{i}\right)-p_{i}^{\alpha }\right)\cdot \left(C_{D}^{w\alpha }\left(v_{j}\right)-p_{j}^{\alpha }\right)}{\lambda^{\alpha }}, \end{array}$$

*where, for k∈{i,j}, $p_{k}^{\alpha }={\left(p+1\right)}^{\left(1-\alpha \right)}\cdot t_{k}^{\alpha }$ with p being the number of triangles, one edge of the triangle is $e_{ij}$, $t_{k}^{\alpha }$ is the weight of the sum of the edges incident to $v_{k}$ that form a triangle with $e_{ij}$ and $\lambda^{\alpha }=\frac{p^{\left(1-\alpha \right)}\cdot {\left(t_{i}+t_{j}\right)}^{\alpha }}{2}+1$.*

**Figure 1:**
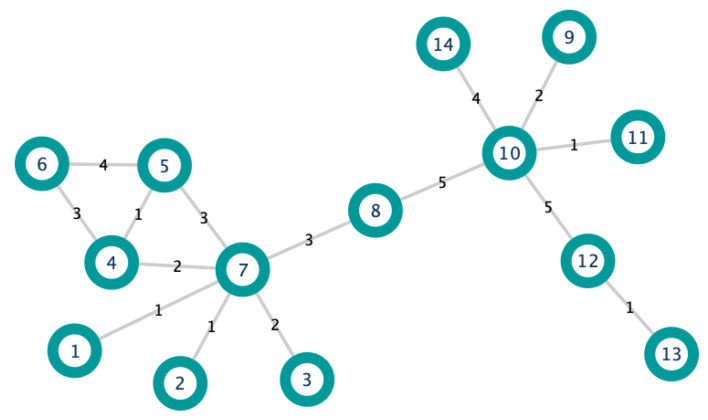
Simple graph.

In order to illustrate the above Definition, let us consider the edge-weighted graph in Figure 1. Moreover, we consider the edges $e_{78}$ and $e_{75}$. Notice that they both have the same weight (three). For this example, we set α=1. Applying Definition 7, we get:

$$\begin{array}{cc} C_{D}^{w1}\left(v_{7}\right) & =deg{\left(v_{7}\right)}^{\left(1-1\right)}\cdot S{\left(v_{t}\right)}^{1}=1\cdot 12=12\;\mathrm{and}\\ C_{D}^{w1}\left(v_{5}\right) & =deg{\left(v_{5}\right)}^{\left(1-1\right)}\cdot S{\left(v_{t}\right)}^{1}=1\cdot 8=8. \end{array}$$

From Definition 8:

$$\begin{array}{cc} p_{7}^{1} & ={\left(1+1\right)}^{\left(1-1\right)}\cdot t_{7}^{\alpha }=2^{0}\cdot 2^{1}=2,\\ p_{5}^{1} & ={\left(1+1\right)}^{\left(1-1\right)}\cdot t_{5}^{\alpha }=2^{0}\cdot 1^{1}=1,\;\mathrm{and}\\ \lambda^{1} & =\frac{1^{\left(1-1\right)}\cdot {\left(2+1\right)}^{1}}{2}+1=\frac{5}{2}. \end{array}$$

Therefore,

$$\begin{array}{cc} I^{1}\left(e_{75}\right) & =\frac{\left(C_{D}^{w1}\left(v_{7}\right)-p_{7}^{1}\right)\cdot \left(C_{D}^{w1}\left(v_{5}\right)-p_{5}^{1}\right)}{\lambda^{1}}\\ \; & =\frac{\left(12-2)\cdot (8-1\right)}{\frac{3}{2}+1}=28. \end{array}$$

In the same way with the edge $e_{78}$, we obtain

$$\begin{array}{cc} I^{1}\left(e_{78}\right) & =96. \end{array}$$

In conclusion, edge $e_{68}$ is more important than edge $e_{75}$.

The latter is reasonable because the edge $e_{78}$ is a bridging edge of the graph.

**Definition** **9** (Contribution [14])**.** *The contribution that $v_{i}\in V$ makes to the importance of the edge $e_{ij}$, denoted by $W^{\alpha }\left(e_{ij}\right)$, is defined as*

$$\begin{array}{cc} W^{\alpha }\left(e_{ij}\right) & =I^{\alpha }\left(e_{ij}\right)\cdot \frac{C_{D}^{w\alpha }\left(v_{i}\right)-w_{ij}^{\alpha }}{C_{D}^{w\alpha }\left(v_{i}\right)+C_{D}^{w\alpha }\left(v_{j}\right)-2w_{ij}^{\alpha }}, \end{array}$$

*where $w_{ij}$ is the weight of $e_{ij}$.*

We have calculated the importance of the edge $e_{78}$ of the graph in Figure 1. The contribution that $v_{7}$ makes to it is given by Definition 9:

$$\begin{array}{cc} W^{1}\left(e_{78}\right) & =I^{1}\left(e_{78}\right)\cdot \frac{C_{D}^{w1}\left(v_{7}\right)-w_{78}^{1}}{C_{D}^{w1}\left(v_{7}\right)+C_{D}^{w1}\left(v_{8}\right)-2w_{78}^{1}}\\ \; & =96\cdot \frac{12-3}{12+8-2\cdot 3}\\ \; & =\frac{432}{7}. \end{array}$$

In the same way, the contribution that $v_{8}$ makes to $I^{1}\left(e_{78}\right)$ is:

$$\begin{array}{cc} W^{1}\left(e_{87}\right) & =I^{1}\left(e_{78}\right)\cdot \frac{C_{D}^{w1}\left(v_{8}\right)-w_{78}^{1}}{C_{D}^{w1}\left(v_{7}\right)+C_{D}^{w1}\left(v_{8}\right)-2w_{78}^{1}}\\ \; & =96\cdot \frac{8-3}{12+8-2\cdot 3}\\ \; & =\frac{240}{7}. \end{array}$$

The above means that the node $v_{7}$ contributes more to the edge $e_{78}$ than node $v_{8}$.

**Definition** **10** (Importance of vertex DIL-W${}^{\alpha }$ [14])**.** *The importance of a vertex $v_{i}\in V$, denoted by $L^{\alpha }\left(v_{i}\right)$, is defined as*

$$\begin{array}{cc} L^{\alpha }\left(v_{i}\right) & =C_{D}^{w\alpha }\left(v_{i}\right)+\sum_{v_{j}\in N\left(v_{i}\right)}W^{\alpha }\left(e_{ij}\right). \end{array}$$

**Remark** **1.** *From the definition of Degree centrality (Definition 7) proposed by Opsahl in [32], we can see that, when the tuning parameter α is* 0*, the Definitions 8–10 are the same than the proposed by Liu et al.*

In order to illustrate the above Definition, we compute the importance of $v_{7}$ and $v_{8}$ in the graph of Figure 1.

$$\begin{array}{ccc} L^{1}\left(v_{7}\right) & = & C_{D}^{w1}\left(v_{7}\right)+\sum_{v_{j}\in N\left(v_{7}\right)}W^{1}\left(e_{7j}\right)\\ & = & 12+W^{1}\left(e_{71}\right)+W^{1}\left(e_{72}\right)+W^{1}\left(e_{73}\right)+W^{1}\left(e_{74}\right)+W^{1}\left(e_{75}\right)+W^{1}\left(e_{78}\right)\\ & = & 12+12+12+24+\frac{75}{7}+18+\frac{432}{7}\\ & = & 78+\frac{507}{7}=\frac{1053}{7}, \end{array}$$

and

$$\begin{array}{ccc} L^{1}\left(v_{8}\right) & = & C_{D}^{w1}\left(v_{8}\right)+\sum_{v_{j}\in N\left(v_{8}\right)}W^{1}\left(e_{8j}\right)\\ & = & 8+W^{1}\left(e_{87}\right)+W^{1}\left(e_{8,10}\right)\\ & = & 8+\frac{240}{7}+\frac{136}{5}\\ & = & 8+\frac{2152}{35}=\frac{2432}{35}. \end{array}$$

Since $L^{1}\left(v_{7}\right)<L^{1}\left(v_{8}\right)$, then node $v_{7}$ is more important than node $v_{8}$ (according to DIL-W${}^{1}$ ranking).

### 2.3. Graph from a Database

The authors of [13] provide a way to obtain an edge-weighted graph from a database, which we briefly detail.

Let $V=\left\{v_{1},v_{2},\ldots ,v_{N}\right\}$ be a set of people registered in a database, denoted by E, with *K* different variables, denoted by $X_{k}$. These variables are separated into two categories: the characteristic variables (CHAR) and the relationship variables (REL) (which are those that allow us to assume that some person meets another). Let us denote by $K_{1}$ the number of relationship variables and EPI(i,k) the response of the person $v_{i}$ to the variable $X_{k}$.

**Definition** **11.** *We will say that a person $v_{i}$ is related to a person $v_{j}$ if and only if there exists $X_{k}\in REL$ for $k\in \{1,2,\ldots ,K_{1}\}$ such that EPI(i,k)=EPI(j,k) and i≠j*.

To define the weight of each link between two persons, we assume that each X∈REL has an associated inherent weight, this is to say, it is possible to discriminate some hierarchical order between the variables. Let $p_{k}$ be the weight associated to the variable $X_{k}\in REL$ for $k=1,\ldots ,K_{1}$.

**Definition** **12.** *We will say that for $X_{j},X_{t}\in REL$, $X_{j}$ is related to $X_{t}$, denoted by $X_{j}RX_{t}$, if and only if $p_{j}=p_{t}$.*

**Definition** **13.** *Let $A_{1},A_{2},\ldots ,A_{c}$ be the different classes that are defined by the different weights $p_{1},p_{2},\ldots ,p_{c}$ and $\alpha_{1},\alpha_{2},\ldots ,\alpha_{c}$ and its respective cardinalities. Hence,*

(2) $$p_{j}=\frac{\alpha_{j}}{K_{1}},$$

*for all j∈{1,2,…,c}.*

We denoted by $h_{i,j}$ the number of times that one person is related to another (or the number of variables that matches between them).

**Definition** **14.** *Let $v_{i},v_{j}\in V$ be such that $v_{i}$ is related to $v_{j}$ and $p_{k_{r}}$ is the weight of the variable in which $v_{i}$ and $v_{j}$ match, for $r=1,\ldots ,h_{i,j}$. We will say that*

(3) $${\tilde{w}}_{ij}=\sum_{r=1}^{h_{i,j}}p_{k_{r}},$$

*is the weight of the link between $v_{i}$ and $v_{j}$.*

Finally, the weighted adjacency matrix, which defines the graph obtained from the database, is the n×n matrix $A\left(G\right)=\left[a_{ij}\right]$, where

$$a_{ij}=\left\{\begin{array}{cc} {\tilde{w}}_{ij} & \mathrm{if}\;\;e_{ij}\in E\left(G\right)\\ 0 & \mathrm{if}\;\;e_{ij}\notin E\left(G\right). \end{array}\right.$$

**Example** **1.** *In the following example, Table 2 simulates a database with 20 registered people. The data hosted correspond to the city in which they live (City), the workplace (considering school and university as a workplace), gender (Gen.), age, extracurricular activity (EC activity), address, whether they drink alcohol (Drin.), whether they are smokers (Sm.) and marital status (MS). Let us consider A and B as two different cities, and x,y,z,w,u,v,r,s,q,t,p,k,d,g and h as different people’s addresses. Moreover, in the table, Y = Yes, N = No, IC = in couple, M = married, S = single, W = widower.*

*From Table 2, we have that $EPI=\{X_{1},X_{2},X_{3},X_{4},X_{5},X_{6},X_{7},X_{8},X_{9}\}$, where $X_{1}=$ City, $X_{2}=$ Workplace, $X_{3}=$ E.P. activity, $X_{4}=$ Address, $X_{5}=$ Sm., $X_{6}=$ Dri., $X_{7}=$ Gen., $X_{8}=$ M.S. and $X_{9}=$ Age. Then, we obtain the sets:*

*1.*  *$REL=\{X_{1},X_{2},X_{3},X_{4}\}$* *and*
*2.*  *$CHAR=\{X_{5},X_{6},X_{7},X_{8},X_{9}\}$*.

*In our criteria, the hierarchical order of the variables $X_{1},X_{2},X_{3},X_{4}$ in descending form is $X_{4},X_{2},X_{3}$, and $X_{1}$. Moreover, we consider that the variables $X_{4}$ and $X_{2}$ have the same weight. Hence, $A_{1}=\left\{X_{2},X_{4}\right\}$, $A_{2}=\left\{X_{3}\right\}$, and $A_{3}=\left\{X_{1}\right\}$ are the different classes that are defined by the different weights. Hence, by Definition 13*

$$p_{1}=\frac{1}{2},\;p_{2}=\frac{1}{4},\;p_{3}=\frac{1}{4}.$$

**Figure 2:**
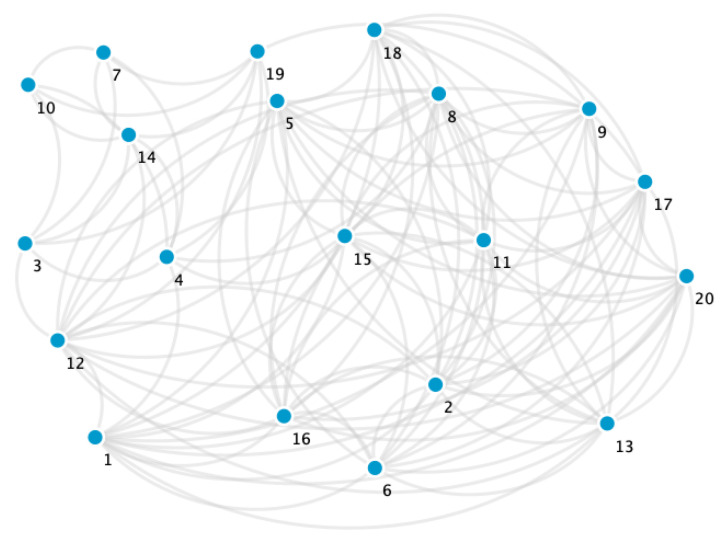
Graph obtained from E.

*To construct the graph, we must resort to Definition 11. For instance, person* 17 *is related to all the people who live in city A or who work at Workplace 8 or who have music as an extra curricular activity or whose address is k. With respect to the weights of the edges, Equation (6) in Definition 14 gives us the answer. For instance, person 6 matches person* 11 *in the answers of the variables $X_{1}$ and $X_{2}$, this is to say, both people live in city A and have the same workplace. Then, the edge $v_{6}v_{11}$ has weight $w_{6\;11}=0.5+0.25=0.75$. Figure 2 shows the obtained graph.*

## 3. Method

The data that are modeled correspond to the city of Olmué (Valparaíso region, Chile) and were obtained from the database of the Epidemiological Surveillance System of the Ministry of Health of Chile, which included the notified cases (positive or negative) and their contacts from 3 March 2020 to 15 January 2021 with a total of 3866 registered persons.

We denote by $E_{pi}$ the database of the Epidemiological Surveillance System of the Ministry of Health of Chile. From the total of variables included in $E_{pi}$ (K=279) 7 of them are relationship variables ($K_{1}=7$). They are: full address ($X_{1}$); the street where the people live ($X_{2}$); town ($X_{3}$); place of work ($X_{4}$); workplace section ($X_{5}$); health facility where they were treated ($X_{6}$) and the region of the country where the test was taken to confirm, or not, the contagion ($X_{7}$).

In our criteria, the hierarchical order of the seven variables in descending form is $X_{1},X_{2},X_{3},X_{4},X_{5},X_{6},X_{7}$. Moreover, we consider that the variables $X_{1},X_{2}$ and $X_{3}$ have the same weight. In the same way, we also consider the variables $X_{4}$ and $X_{5}$ with equal weight. Hence, $A_{1}=\left\{X_{1},X_{2},X_{3}\right\}$, $A_{2}=\left\{X_{4},X_{5}\right\}$, $A_{3}=\left\{X_{6}\right\}$ and $A_{4}=\left\{X_{7}\right\}$ are the different classes that are defined by the different weights. Hence, by Definition 13.

$$p_{1}=\frac{3}{7},\;p_{2}=\frac{2}{7},\;p_{3}=\frac{1}{7},\;p_{4}=\frac{1}{7}.$$

**Figure 3:**
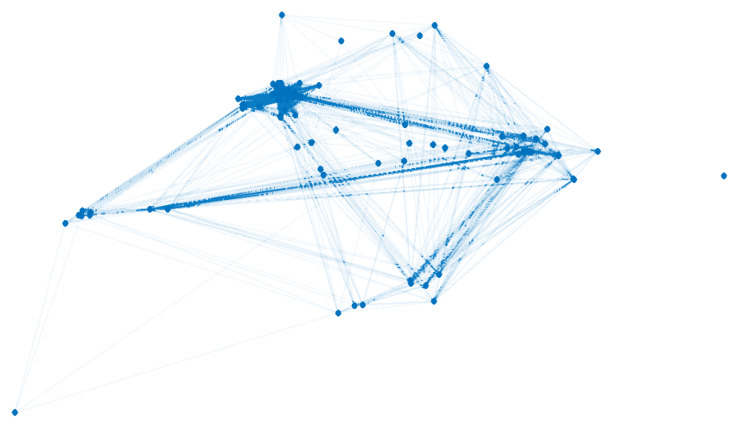
Graph obtained from database of Olmué city, Chile, with 3866 vertices and 6,841,470 edges.

Figure 3 shows the obtained graph.

Let us denote by $G_{E}$ the graph obtained from database $E_{pi}$.

### Strategy Protection

In this section, we provide definitions of the protection of a graph when disease spreads on it. Moreover, we state the protection strategy used in the graph $G_{E}$ obtained in the previous Section. The following definitions come from [28].

**Figure 4:**
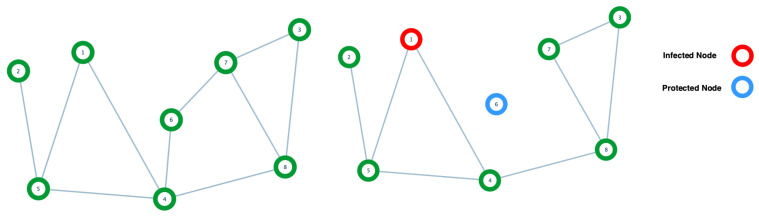
The left side shows a graph without protection or infection. On the right side, we see an infected node (red) and a protected node (blue) in the same graph.

**Definition** **15.** *Protecting a vertex means removing all of its corresponding edges. (See Figure 4).*

It is also possible to find in the literature that protecting a vertex means removing the vertex from the graph. See, for instance, [20].

**Definition** **16.** *The number of vertices that are allowed to protect is called the protection budget, denoted by k.*

**Definition** **17.** *We will say that the survival rate, denoted by σ, is the ratio of vertices that remain uninfected at the end of the disease over the total numbers of vertices.*

Therefore, our problem is: given a graph G=(E,V), SIR model, and a protection budget *k*, the goal is to find a set of vertices S⊆V, such that

(4) $$\begin{array}{cc} \theta^{*} & =\arg\max_{S\subseteq V}\sigma , \end{array}$$

with |S|=k. However, the problem (4) is NP-Hard (see [34]).

Our chosen protection strategy corresponds to the DIL-W${}^{\alpha }$ ranking (see [14]). It is well known that an index to measure the connection of a graph is the efficiency of the networks (see [35]). High connectivity of the graph indicates high efficiency. In [14], the authors show that the DIL-W${}^{\alpha }$ ranking provides good results regarding the rate of decline in network efficiency (for more detail see [36]), when it comes to eliminating the best positioned nodes by this ranking. One of the good qualities of the DIL-W${}^{\alpha }$ ranking is that it recognizes the importance of bridge nodes (see more in [37]). This quality is inherited from the version of the DIL ranking for graphs not weighted at the edges (see [33]). Furthermore, [28] evaluated the effectiveness of the DIL-W${}^{\alpha }$ ranking in the immunization of nodes that are attacked by an infectious disease that spreads on an edge-weighted graph using a graph-based SIR model.

**Figure 5:**
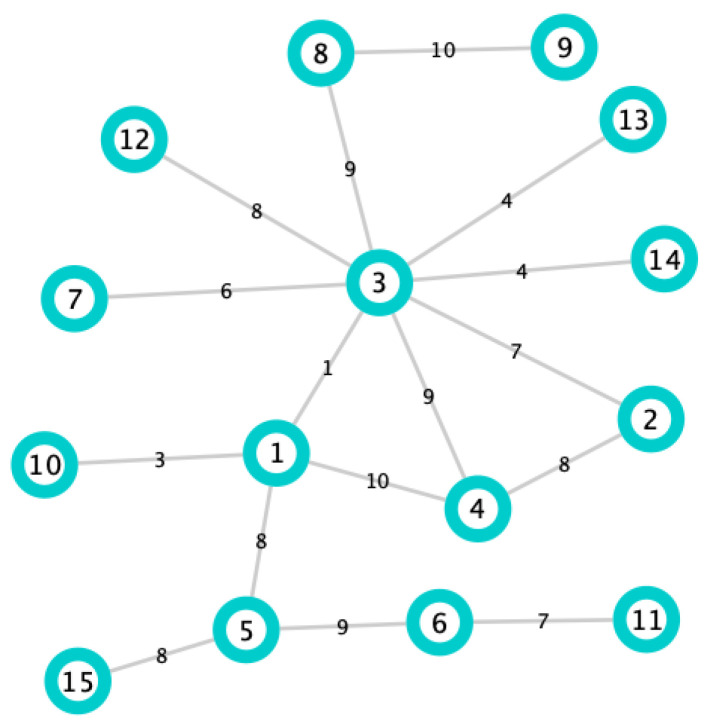
Graph with 15 nodes, 16 edges, and the respective weights.

**Figure 6:**
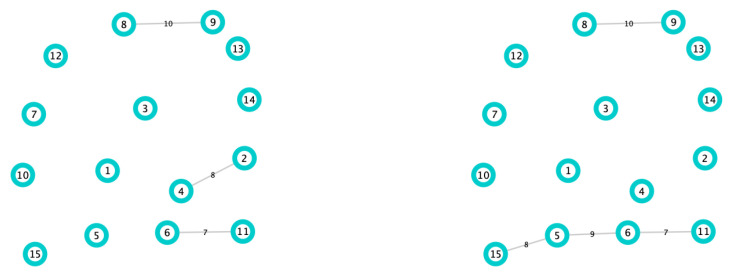
In the left, the first 3 protected places generated by the DIL-W${}^{1}$ ranking. On the right, the first 3 protected places generated by Strength ranking.

Finally, in order to illustrate in a simple way why the DIL-W${}^{\alpha }$ ranking has been chosen, let us consider the graph of Figure 5 with 16 edges, 15 nodes, and the respective weights on the edges. When we apply the DIL-W${}^{1}$ ranking, the first 3 places are occupied by nodes 3, 5 and 1, respectively. These nodes are precise bridge nodes and, when protecting them, according to Definition 15, the graph loses connectivity (see Figure 6). If we apply the Strength ranking, the first three places are occupied by nodes 3, 1 and 4, respectively. Note that the order in which it positions the nodes and the importance it gives to node 4 makes the loss of network connectivity lower than the loss when applying DIL-W${}^{1}$ (see Figure 6).

In summary, we apply the protection to the $G_{E}$ network, according to the importance ranking list produced by DIL-W${}^{\alpha }$, considering different protection budgets. For the ranking DIL-W${}^{\alpha }$, we consider three different values for α; they are 0, 0.5 and 1. In this way, we compare how the protection performs according to the value of α.

## 4. Results

In this paper, we use a graph-based SIR model in the same way as in [13,28], namely, each individual is represented by a vertex in $G_{E}$. At time *t*, each vertex $v_{i}$ is in a state $v_{i}^{t}$ belonging to S={0,1,−1}, where 0,1 and −1 represent the three discrete states: Susceptible (S), Infected (I) and Recovered or Removed (R). We set

(5) $$N_{I}\left(v_{i}\right)=\left\{v\in N(v_{i}):v\in I\right\}.$$

At time t+Δt, the vertex $v_{i}$ will change state according to probabilistic rules:

1. The probability ($P_{I}\left(v_{i}\right)$) that a susceptible vertex $v_{i}$ is infected by one of its neighbors is given by
  (6) $$P_{I}\left(v_{i}\right)=\sum_{v_{j}\in N_{I}\left(v_{i}\right)}\rho \Delta t\cdot w_{ij},$$
  where ρ is a purely biological factor and representative of the disease and $w_{ij}$ is the weight of the edge $e_{ij}$.
2. The probability ($P_{R}\left(v_{i}\right)$) that an infected vertex $v_{i}$ at time *t* will recover is given by
  (7) $$P_{R}\left(v_{i}\right)=\delta \Delta t,$$
  where δ is the recovery rate.

Moreover, we assume that the disease is present for a certain period of time and that, when individuals recover, they are immune, that is, reinfection is not considered.

**Figure 7:**
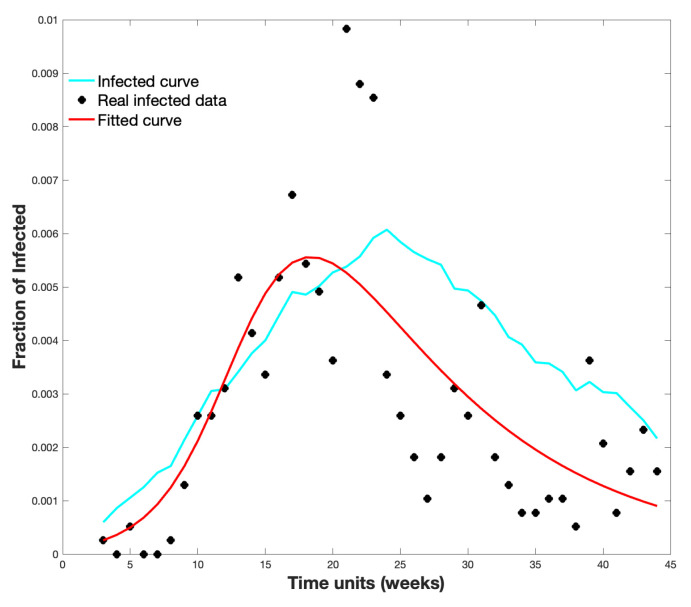
Real infected data (black), fitted curve (red), and infected curve obtained in the spread on $G_{E}$ (cyan).

The initial population contains one infected node and all the simulations that consider $\delta =\frac{1}{15}$. Five hundred simulations were performed on $G_{E}$ with ρ=0.00121. Figure 7 shows the average infected curve and the real infected data in $E_{pi}$. Moreover, it shows a curve fitted to the data following the SIR model; for this, we used the classic method of least squares to compare with our proposal.

**Figure 8:**
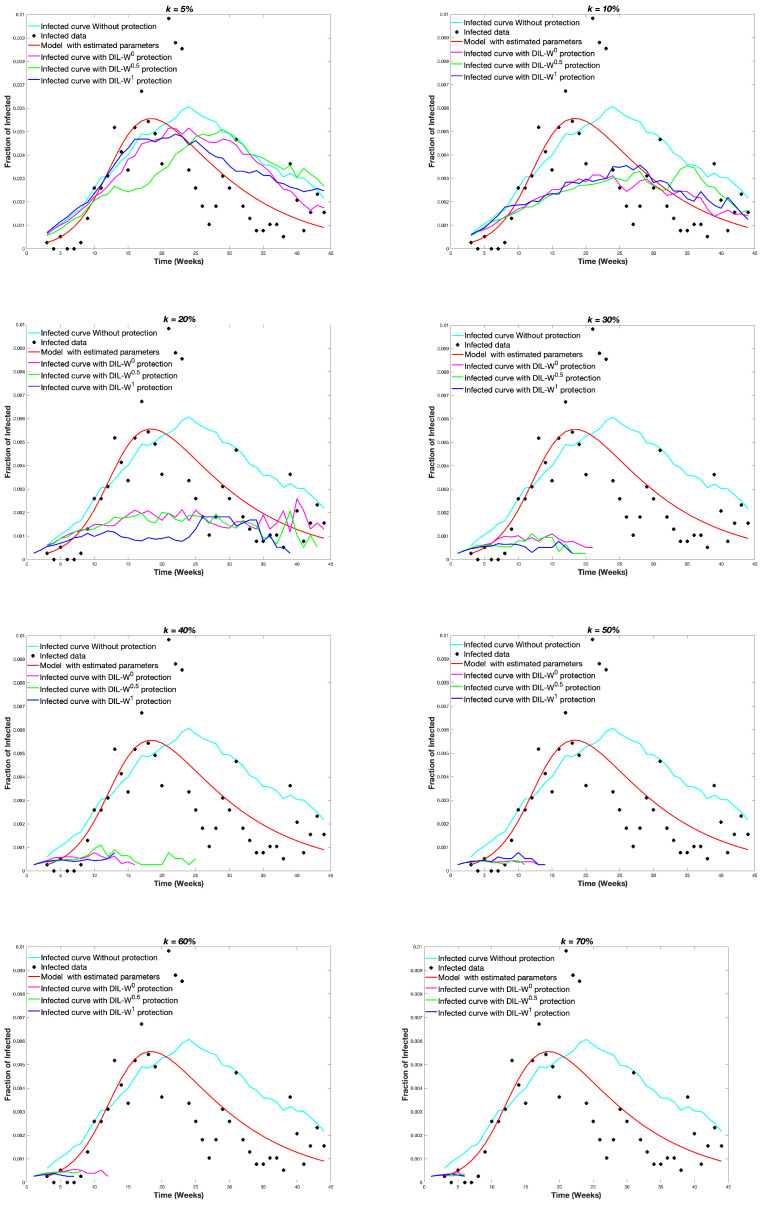
Infected curves obtained according to different values of *k*.

The graph $G_{E}$ was protected with different protection budgets according to the importance of the DIL-W${}^{0}$, DIL-W${}^{0.5}$ and DIL-W${}^{1}$ rankings. Protection is carried out in week 1, this is to say, at the beginning of the spread of the disease. Figure 8 shows the results.

**Figure 9:**
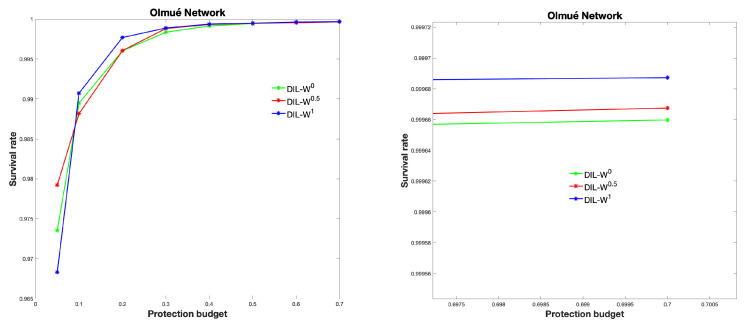
Survival rate of each ranking.

We can see the survival rate in Figure 9.

**Figure 10:**
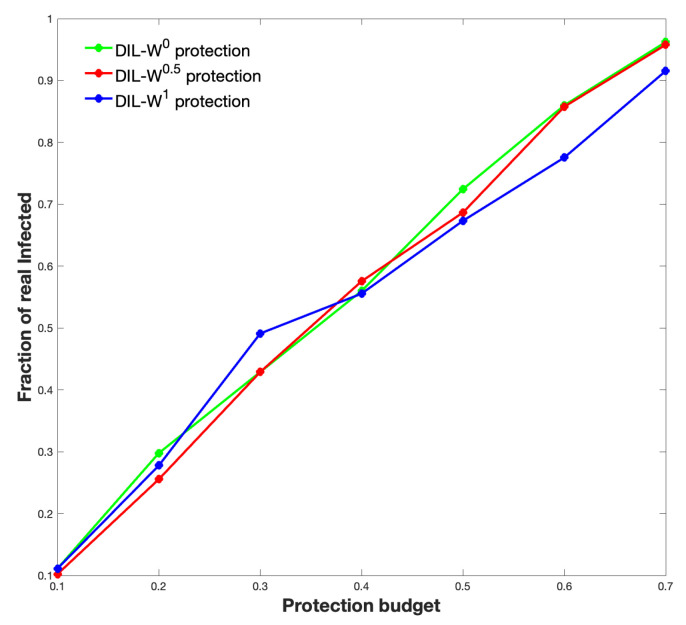
Relationship between the real infected and those immunized according to the different DIL-W${}^{\alpha }$ ranking.

Figure 10 shows the relationship between the real infected (450 people) and those immunized according to our proposal.

We can see that 80% of the real infected are located in 60% of the top ranked according to DIL-W${}^{\alpha }$. We think that this is a way to recognize those who will get sick; however, it is not the solution.

**Figure 11:**
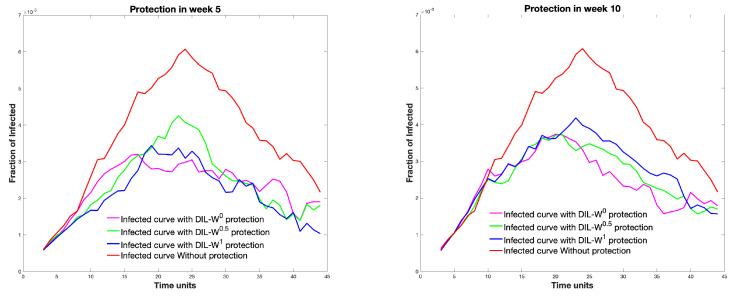

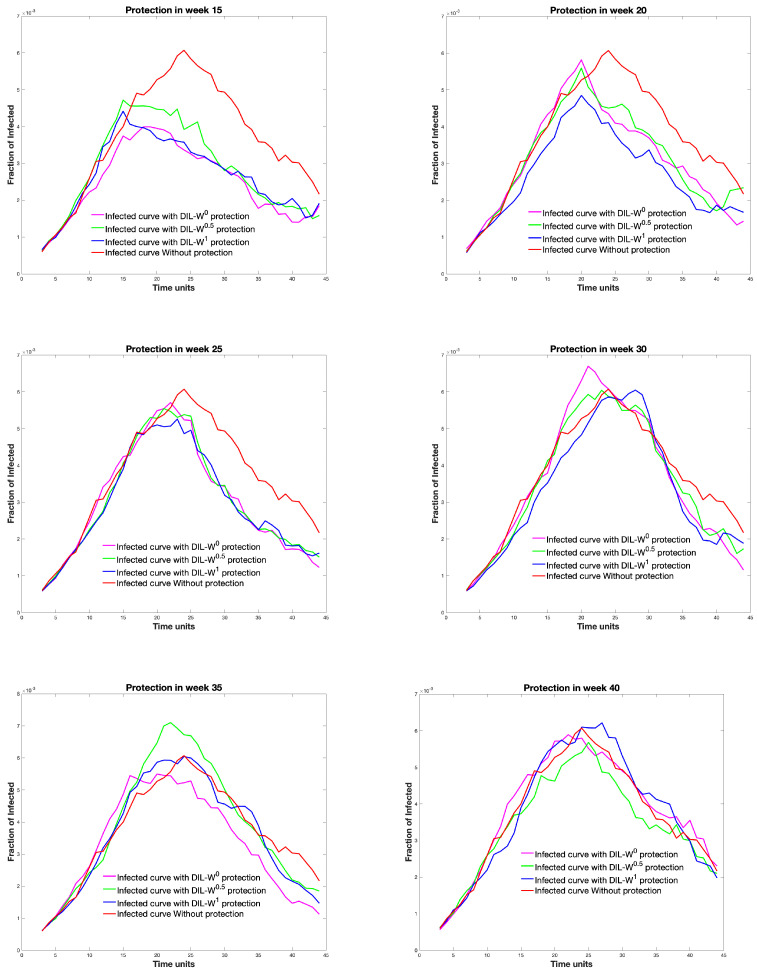
The different infected curves, considering the 10% protection according to the DIL-Wαranking and carried out in different weeks.

Another element that we have considered investigating is the time at which the protection takes place. We modified the protection in the graph as the weeks advanced. In Figure 11, we can see the different infected curves, considering the 10% protection according to the DIL-W${}^{\alpha }$ ranking.

**Figure 12:**
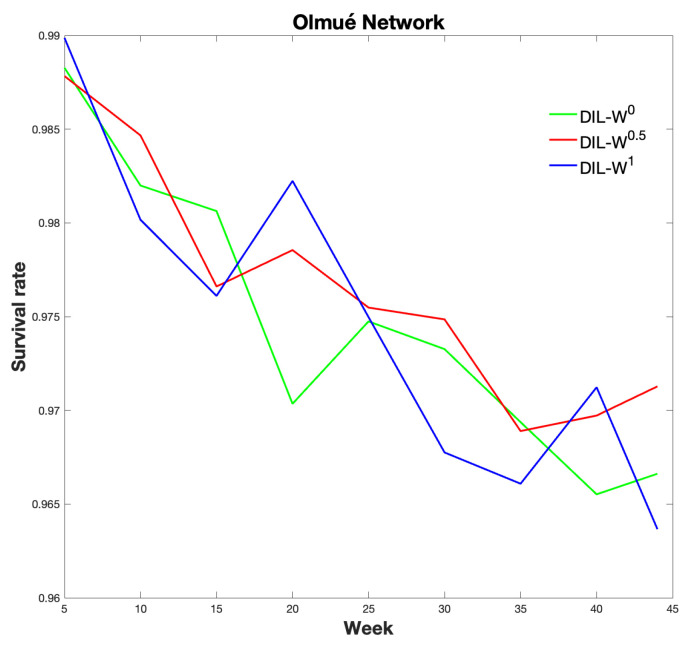
Survival rate of each ranking.

Figure 12 shows the relationship between the survival rate and the week in which the protection is carried out with our proposal.

The survival rate is clearly decreasing.

## 5. Discussion

The results of the present investigation are directed towards the analysis of the effectiveness of immunization using the DIL-W${}^{\alpha }$ ranking with real COVID-19 data from the city of Olmué-in Chile. Depending on the importance of the rankings, the immunization results were similar, despite the percentage of protection proposed in the simulations. Our method, therefore, goes in the direction of finding new optimization algorithms in network protection strategies [38].

At the level of protection, it is evident that when the percentage of initial coverage is higher, the epidemic ends with a smaller fraction of people affected by COVID-19. This event is related not only to the random increase in immunization, but also to the possibility, in the model, of recognizing the bridge nodes to increase the effectiveness of vaccination. This is consistent with other investigations that indicate that the recognition of central nodes or high-risk individuals improves the efficiency of immunization strategies in real networks, a situation that favors the protection of the network and the best use of vaccine doses [39,40]. The best use of doses is a challenge for the current scenario of vaccine shortages worldwide, mainly in poor nations [41].

On the other hand, the level of effectiveness of the DIL-W${}^{\alpha }$ ranking, given the percentage of protection, is established in the recognition of the bridging nodes in a regular vaccination process. The results using the α parameters of DIL-W${}^{\alpha }$ indicate a high survival rate. DIL-W${}^{1}$ achieves better results with 70% protection and is positioned with the best survival rate, but DIL-W${}^{0}$ and DIL-W${}^{0.5}$ show good results. The difference between the different values of α is marginal and can be explained by the adequate representation that the DIL-W${}^{\alpha }$ model has and by the values of α, which do not generate excessive differences in the ranking. This is similar to the results of the research by Ophsal et al. who, through Freeman’s EIES network, mention that the centrality degree (Definition 7) is relatively stable among the different α parameters [32].

In a real and regular immunization strategy situation, such as the administration of vaccines, determining the population that infects most frequently is relevant since it allows optimization of these processes. Among our findings, it stands out that 80% of the real infected in the Olmué-Chile commune were located in 60% of the top of the DIL-W${}^{\alpha }$ ranking. Consequently, our proposal recognizes the heterogeneity of the network, approaching the reality of human interactions and achieving similar results in complex homogeneous networks [40].

Regarding immunization with 10% protection, a decrease in the survival rate is established by 4% from weeks 5 to 45 of protection. Likewise, with the same percentage of protection, the effectiveness of the immunity strategy tends to be important until week 20. After 20 weeks, the fraction of infected is similar with or without protection. Consequently, our model is strongly effective as a measure of rapid recognition of the epidemic outbreak in a given territory.

Therefore, according to the findings of our research, there are two important variables for the success and effectiveness of immunity strategies against COVID-19: (1) Recognition of bridging nodes (people with the highest probability of contagion) to apply measures of protection; and (2) the development time of this strategy.

Regarding the recognition of bridging nodes, there is evidence to support that targeted immunization schemes significantly reduce epidemic outbreaks [42]. This opens the possibility of changing the traditional perspective of immunization by protecting a small proportion of the population over a long period of time [43]. It is important, therefore, not only to direct COVID-19 immunity efforts towards the population most affected by mortality, but also in those population groups that tend to infect with greater force.

The time of development of the immunization strategy continues to be a variable under discussion in the scientific community regarding the slowness worldwide of the vaccination process, which risks not achieving herd immunity [44]. In summary, both at a theoretical and empirical level, the execution time of immunization strategies is important in overcoming the COVID-19 pandemic.

Finally, our model helps to establish a ranking of bridge nodes in a non-homogeneous network, so it is highly replicable with real COVID-19 dissemination data and it is useful to establish more focused strategies given the reduced number of vaccines available.

## 6. Conclusions

In this paper, we evaluate the effectiveness of the DIL-W${}^{\alpha }$ ranking in the immunization of nodes that are attacked by an infectious disease (COVID-19) that spreads on an edge-weighted graph obtained from the database of the Epidemiological Surveillance System of the Chilean Ministry of Health, using a graph-based SIR model.

Considering survival rates, the DIL-W${}^{1}$ ranking performs better (by a small margin) than DIL-W${}^{0.5}$ and DIL-W${}^{0}$ rankings, subject to the protection budget being equal to 10% of the network nodes.

The period in which immunization or protection is given plays a key role in stopping the spread of the disease (see Figure 11) since around week 25 immunization does not generate a great impact and as time progresses the survival rate decreases almost linearly.

An interesting and complex task to solve is to determine which value of α to choose in the network so that the ranking generated is the optimal one. The same value does not always make the performance the best. One way to explore this is to continue with the ideas proposed in [45], where the selection standard of the optimal turning parameters is proposed for the centrality degree, but is not for DIL-W${}^{\alpha }$ ranking. However, when considering this method, there are as many rankings as there are numbers between 0 and 1.

## Figures and Tables

**Table 1:** Summary of Symbols and Notations.

Notations	Definition and Description
*G*	Graph or network.
(G,w)	Edge-weighted graph.
$v_{i}$	Vertex or node.
$N(v_{i})$	Neighborhood of a vertex *v*.
$e_{ij}$	Edge between vertex $v_{i}$ and vertex $v_{j}$.
$w_{ij}$	Weight of the edge $e_{ij}$.
$deg(v_{i})$	Degree of the vertex $v_{i}$.
$S(v_{i})$	Strength of the vertex $v_{i}$.
α	Real number. Tuning parameter.
$C_{D}^{w\alpha }\left(v_{i}\right)$	Degree centrality of $v_{i}\in V$ of an edge-weighted graph (G,w).
DIL-W${}^{\alpha }$	Ranking based on Degree and importance of line.
$I^{\alpha }\left(e_{ij}\right)$	Importance of edge $e_{ij}$.
$W^{\alpha }\left(e_{ij}\right)$	Contribution that $v_{i}$ makes to the importance of the edge $e_{ij}$.
$L^{\alpha }\left(v_{i}\right)$	The importance of a vertex $v_{i}$.
E	Database.
$X_{k}$	Variable of a database.
$p_{k}$	Weight of the variable $X_{k}$.
*k*	Protection budget (the number of nodes in graph *G* that can be protected).
σ	Ratio of surviving nodes.

**Table 2:** Database E.

Person	City	Workplace	E. C. Activity	Address	Sm.	Dri.	Gen.	M. S	Age
1	A	Workplace 1	Theater	y	Y	Y	F	IC	35
2	A	Workplace 3	Cinema	y	Y	Y	M	IC	35
3	B	School B	Football	z	N	N	F	S	10
4	B	Workplace 1	Photography	x	N	N	F	M	48
5	A	Workplace 5	Does not have	u	Y	N	F	W	65
6	A	Workplace 4	Does not have	v	Y	Y	M	IC	27
7	B	Workplace 2	Does not have	x	Y	N	M	M	46
8	A	University 1	Photography	v	N	N	M	IC	29
9	A	University 2	Does not have	w	Y	Y	M	IC	19
10	B	School B	Karate	x	N	N	M	S	10
11	A	Workplace 4	Ping-pong	r	Y	Y	F	M	54
12	A	School A	Football	s	N	N	M	S	8
13	A	Workplace 5	Dance	r	Y	Y	F	M	57
14	B	School A	Handball	q	N	N	M	S	11
15	A	University 1	Does not have	t	N	N	F	S	25
16	A	Workplace 7	Singing	p	Y	Y	F	S	60
17	A	Workplace 8	Music	k	N	Y	F	S	28
18	A	Workplace 3	Does not have	d	N	N	M	S	47
19	B	School A	Music	g	N	N	F	S	8
20	A	Workplace 6	Does not have	h	Y	Y	M	S	30

## Data Availability

The data presented in this study were available after being requested by research project COVID-ANID to the Chilean Ministry of Health. The data are not publicly available due to legal restrictions. The source codes are accessible through the Github link: https://github.com/RonaldManriquez/Prot-stra-aga-epi-ewgraphs.git, from 8 June 2021.
